# Report of a patient and further clinical and molecular characterization of interstitial 4p16.3 microduplication

**DOI:** 10.1186/s13039-015-0119-6

**Published:** 2015-02-28

**Authors:** Orazio Palumbo, Pietro Palumbo, Emanuela Ferri, Francesco Nicola Riviello, Lea Cloroformio, Massimo Carella, Marilena Carmela Di Giacomo

**Affiliations:** Laboratorio di Genetica Medica, IRCCS Casa Sollievo della Sofferenza, San Giovanni Rotondo, FG Italy; U.O.C Anatomia Patologica, AOR Ospedale “San Carlo”, Potenza, Italy

**Keywords:** 4p16.3 duplication, SNP array analysis, Genotype-phenotype correlation

## Abstract

**Background:**

Pure interstitial duplications of chromosome band 4p16.3 represent an infrequent chromosomal finding with, to the best of our knowledge, only two patients to date reported.

**Case presentation:**

We report on a 13-year-old boy showing a set of dysmorphic facial features, attention deficit hyperactivity disorders, learning difficulties, speech and cognitive delays, overgrowth and musculoskeletal anomalies in whom an interstitial duplication of about 400 kb in 4p16.3 was detected by SNP-array analysis. The duplication includes the complete coding sequence of *FAM53A*, *SLBP*, *TMEM129* and *TACC3* genes and the first exon of the *FGFR3* gene. Phenotypic comparison with previously described patients harboring a microduplication of similar size and position contributes to better define the clinical correlation of 4p16.3 microduplications, suggesting the existence of a novel distinct and phenotypically recognizable syndrome. In addition, being the duplication identified in our case the smallest so far reported, it allowed us to refine the smallest region of overlap among patients to 222 kb, enabling a more accurate genotype-phenotype correlation for 4p16.3 microduplications.

**Conclusions:**

Our case report provide clinical and molecular evidences supporting the existence of a novel 4p16.3 microduplication syndrome. The genes *FAM53A*, *TACC3* and *FGFR3* seems to play a key role in the etiology of the clinical phenotype*.* Interestingly, our patient is the oldest described so far and for this reason useful to delineate the long-term prognosis of these patients*.*

## Background

Small (<1 Mb in size) and pure interstitial microduplications of the distal short arm of chromosome 4 are rare; to the best of our knowledge, only two patients have been to date reported. Some years ago, Hannes et al. [1] described a 23-month-old boy whit neurodevelopmental delay, seizures, glaucoma of the left eye and dysmorphic features in whom a de novo submicroscopic (560 kb) duplication of 4p involving the Wolf-Hirschhorn critical region (WHSCR) has been identified. Later, Cyr at al. [2] using a high-density oligonucleotide microarray described the first patient carriers of a de novo 506 kb microduplication in 4p16.3, distal to WHSCR, showing developmental delay, seizures, macrocephaly, unilateral glaucoma, abnormal hands and dysmorphic features.

In this study, we report an additional patient with the smallest overlapping duplication in 4p16.3, detected by SNP-array analysis, not including the WHSCR delineating the duplications’ phenotype. In addition, refinement of the smallest region of overlap (SRO) to 222 kb highlights interesting genes as candidates for the common observed clinical features, suggesting that the duplication in 4p16.3, distal to WHSCR, may represent a novel clinically recognizable condition.

## Case presentation

### Case report

**Figure 1:**
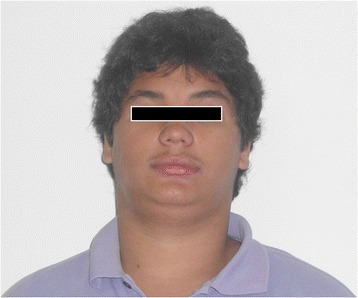
**The picture of the proband at the age of 13 years.**

The male patient was referred for the first time at the age of 10 years for evaluation of autistic features, learning difficulties, speech and cognitive delays. No information are available on family history since he was adopted. Physical examination at 13 years of age revealed auxological parameters above the average (weight >97th centile; height between the 90th and 97th centile) and dysmorphic features including high forehead with frontal bossing, small palpebral fissures, epicanthal folds, hypertelorism (>2 SD), dental abnormalities, high arched palate, micrognathia, short neck (Figure 1). In addition, hyperopia and skeletal anomalies (scoliosis), gynecomastia and bilateral pes planus were reported.

Neuropsychiatric evaluation showed speech delay, mild cognitive impairment (QI 68), attention deficit hyperactivity disorder (ADHD). Also, the patient showed walking abnormalities (uncertain gait), clumsiness.

Abdominal ultrasound examination and brain magnetic resonance imaging (MRI) were normal. Genetic evaluation revealed a normal 46,XY karyotype while DNA analysis for *FMR-1* gene excluded the diagnosis of fragile X (FRAXA) syndrome.

## Results

SNP array analysis showed a 4p16.3 duplication of a minimum size of 393 kb, from nucleotide nt 1,405,662 (first duplicated probe: CN_1080836) to nucleotide nt 1,798,461 (last duplicated probe: CN_1081687), and a maximum size of 413 kb, from nucleotide nt 1,390,388 (last present probe before the duplication: CN_1069802) to nucleotide 1,804,276 (first present probe after the duplication: SNP_A-2213611).

The duplicated region encompass the entire coding sequences of *FAM53A*, *SLBP*, *TMEM129*, *TACC3* genes and the 5′ end of the *FGFR3* gene (data not shown). According to the International System for Human Cytogenetic Nomenclature (ISCN) 2013, molecular karyotype of the patient was arr[hg19]4p16.3(1,405,662-1,798,461)×3. There were no other clinically significant genomic alterations identified.

## Discussion

**Figure 2:**
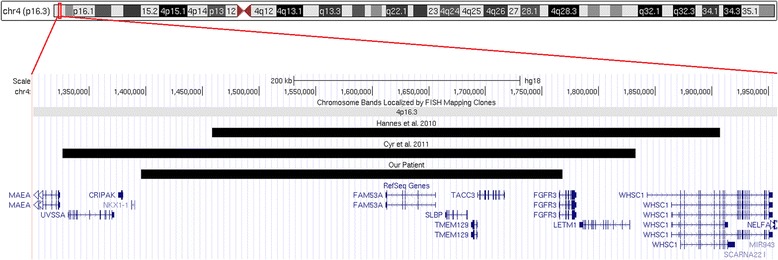
**Schematic representation of the 4p16.3 duplications in the present and previously reported patients based on the UCSC genome browser 2009 assembly (GRCh37/hg19) [** **http://genome.ucsc.edu/** **].**

We report on a 13-year-old boy with an interstitial 393 kb duplication on the short arm of chromosome 4 (4p16.3). To our knowledge, there are only two patients reported in the literature carriers of a duplications similar in size and position to the one identified in our patients [1,2]. The clinical phenotype of our patient, similarities and differences with previously reported cases are discussed in detail and summarized in Table 1 while the location of the duplications is shown in Figure 2. The clinical features shared by all three individuals include psychomotor and language delay, musculoskeletal anomalies, eyes alterations as well as craniofacial anomalies (high forehead, frontal bossing, epicanthal folds, hypertelorism/abnormal palpebral fissures, short neck) suggesting that the cryptic 4p16.3 duplications results in a novel recognizable microduplication syndrome. Seizure, MRI anomalies, abnormal ears, high arched palate and growth alterations (macrocephaly in the patient reported by Cyr et al., overgrowth in our) are also significant issues in these patients (2/3). Others phenotypes observed are hypotonia in the patient reported by Hannes et al. [1], mild intellectual disability and micrognathia in our case. Some differences in clinical presentations among the 4p16.3 microduplicated patients could be explained by the influence of the genetic background of the rest of the genome and/or the variable size and gene content of the rearrangements. These factors could have additional or modifying influences on the clinical features.

**Table 1:** **Summary of the clinical features of the patients with 4p16.3 duplication overlapping with our patient**

**Clinical features**	**Present case**	**Hannes et al.** [1]	**Cyr et al.** [2]
**Sex and age at diagnosis**	M, 13 years	M, 23 months	M, 9 months
Weight	>97th centile	< 3rd centile	30th centile
Height	90-97th centile	N.R.	30th centile
Head circumference	25th-50th centile	N.R.	>95th centile
**Neurocognitive**	Mild ID (IQ 68)	Unknown	Unknown
**Neurologic**	Delayed motor development and speech	Delayed motor development and speech, seizure, hypotonia	Delayed motor development, seizure
**Neuropsychiatric**	ADHD	N.R.	Too young
**MRI**	Normal	Delayed myelinisation	Dilatation of the lateral ventricles
**Craniofacial**			
Macro/Microcephaly	N.R.	Unknown	Macrocephaly
*Forehead*	High	High	High
Frontal bossing	+	+	+
Epicanthal folds	+	+	+
Hypertelorism	+	-	+
Abnormal palpebral fissures	Narrow and long	Narrow and long	Downslanted
Nose	Normal	Normal	Broad nasal root and short nasal bridge
Low set/abnormal ears	Normal	Low-set and dysmorphic	Low-set and posteriorly rotated
Palate	High arched	High arched	Normal
Micrognathia	Present	Absent	Absent
Neck	Short	Short	Short
**Musculoskeletal**	Scoliosis, bilateral flatfoot	Small hands and feet, malformations of the right hand	N.R.
**Others**	Hyperopia, dental abnormalities, gynecomastia	Glaucoma (left eye), hypoacusis of the right ear	Irregular iris pigmentation-heterochromia, hyperopia

+, present; −, absent; ID, intellectual disability; ADHD, attention deficit hyperactivity disorder; **N.R.**, not reported.

At a molecular level the small size (393 kb) of the duplication we present here allowed us to identify the SRO among the patients and focus our attention on interesting candidate genes that could be associated to the common traits reported.

The minimal duplicated region shared by all three patients is about 222 kb (1,575,789-1,798,461 bp), has never been reported as copy number polymorphism (CNP) in the Database of Genomic Variant and encompass the entire sequence of the genes *FAM53A*, *SLBP*, *TMEM129*, *TACC3* and the 5′ end of the *FGFR3* gene. Of these genes, *FAM53A* and *TACC3* are the best candidate for the neurodevelopmental features because highly expressed in the early embryonic central nervous system (FAM53A) suggesting critical roles in the neuronal development [3] or encoding a protein (TACC3) that controls the genesis of neurons from radial glial progenitor cells (RGCs) during cortical development [4]. In addition, mutations of the murine Tacc3 leads to retarded growth, apoptosis of hematopoietic stem cells and facial clefting [5]. The apparent role in facial development makes this gene of interest also for the craniofacial traits of the 4p16.3 microduplicated patients discussed: some of the affected tissues are equivalent to those that are dysgenic in the patients (i.e. the tissues derived from the fronto-nasal mass). Although these evidences suggest that *TACC3* could be responsible for the dysmorphic facial features reported, functional studies, additional experimental and clinical data are needed to corroborate our hypothesis.

Regarding the growth alterations and the musculoskeletal malformations the best candidate is the gene *FGFR3* (fibroblast growth factor receptor 3) that is a regulator of bone growth. Mutations in this gene has been associated with at least ten human disorders where skeletal alterations represent the principal clinical presentations. It is interesting to highlight the fact that the features concerning the growth (overgrowth in our patient), or at least some of its parameters (macrocephaly in the patient described by Cyr et al.), of patients with duplication of *FGFR3* are opposed to that of Wolf-Hirschhorn Syndrome (WHS; del4p16.3) patients (microcephaly, growth retardation). This phenomenon supports the hypothesis of mirror phenotypes resulting from reciprocal deletion/duplication in chromosomal regions containing dosage sensitive genes as described for other copy number variants (16q11.2, 7q11.23, 5q35) [6,7].

In all patients to date reported, eyes anomalies have been documented such as glaucoma in the patient described in the Hannes’ paper, the iris pigmentation- heterochromia reported by Cyr et al. and the hyperopia of our patient. Since this clinical evidence, in agreement with Cyr et al., we consider the 4p16.3 locus very important in ocular development although, at this time, we cannot be able to explain the biological mechanism related to 4p16.3 trisomy underlying these alterations. Evaluation of more patients is needed to clarify this point.

Our patient does not share the seizures that are present in the other two individuals. In contrast to the 4p16.3 duplications described by Hannes et al. [1] and Cyr et al. [2] the duplication identified in the present patient do not encompass the *LETM1* gene supporting the hypothesis that overexpression of this gene may results in seizure [2].

Needs to be elucidated the involvement of the *SLBP* and *TMEM129* genes in the etiology of the phenotype in patients with dup4p16.3. The first encodes an RNA-binding protein that recognize a stem-loop structure in 3′ non-coding sequence of histone mRNAs essential for maturational cleavage and efficient translation of the encode histone [8] while the function of the latter remains to be explored. Their contribution to the phenotype, if any, is unknown*.* Evaluation of additional patients with well-characterized 4p16.3 duplication and/or point mutations in this region will be useful to elucidate the role of individual genes for the clinical presentations.

Of note, our patients is the only one in which a consistent pattern of neurobehavioral features including ADHD, has been documented. Since the other two patients are too young, we cannot exclude that they will show behavioral alterations in the feature thus we suggest a periodic neurobehavioral clinical evaluation in 4p16.3 microduplication carriers.

## Conclusions

In conclusion, in this report we provide clinical and molecular evidences supporting the existence of a novel 4p16.3 microduplication syndrome. The shared clinical features between our patient and those in previous reports include psychomotor and language delay, skeletal anomalies and a particular pattern of facial dysmorphisms. Narrowing the SRO to include only five genes we performed a more detailed genotype-phenotype correlation suggesting the *FAM53A, TACC3* and *FGFR3* genes as candidates for the clinical manifestation of this syndrome. Finally, being our patients the oldest reported so far he provides detailed clinical and phenotype informations across the lifespan facilitating a more accurate genetic counselling and anticipatory care.

## Methods

### SNP array analysis

Total DNA was obtained from peripheral blood using automated BioRobot EZ1 (Qiagen, Solna, Sweden). We checked it for quantity and purity using the NanoDrop ND-1000 Spectrophotometer (Thermo Fisher Scientific, Wilmington, DE). Molecular karyotyping was performed using the high-resolution Genome Wide Human SNP Array 6.0 (Affymetrix, Santa Clara, CA) providing whole genome coverage with a density of 1.8 million markers, including 906,600 SNPs and 946,000 copy number markers. Labeling, hybridization, washing, scanning and image extraction were performed as previously described [9]. Copy number variations (CNVs) identified in this study and overlapping with annotated CNVs in Database of Genomic Variants (DGV; http://dgv.tcag.ca/dgv/app/home), were not considered. Furthermore, we filtered all CNVs that had ≤50 contributing markers.

## Consent

We obtained written informed consent from the patient for publication of this Case report and any accompanying images. A copy of the written consent is available for review by the Editor of this journal.

## Abbreviations

WHSCR: Wolf-Hirschhorn critical region
SRO: Smallest region of overlap
ADHD: Attention deficit hyperactivity disorder
MRI: Magnetic resonance imaging
CNVs: Copy number variations
DGV: Database of Genomic Variants
ISCN: International System for Human Cytogenetic Nomenclature
CNP: Copy number polymorphism
RGCs: Radial glial progenitor cells
*FGFR3*: Fibroblast growth factor receptor 3
